# 3D correction of AIS in braces designed using CAD/CAM and FEM: a randomized controlled trial

**DOI:** 10.1186/s13013-017-0128-9

**Published:** 2017-07-23

**Authors:** Nikita Cobetto, Carl-Éric Aubin, Stefan Parent, Soraya Barchi, Isabelle Turgeon, Hubert Labelle

**Affiliations:** Department of Mechanical Engineering, Polytechnique Montreal, P.O. Box 6079, Downtown Station, Montreal, Quebec H3C 3A7 Canada

**Keywords:** Computer-aided design/computer-aided manufacturing, Scoliosis, Thoraco-lumbo-sacral orthosis, Finite element model (FEM), RCT

## Abstract

**Background:**

Recent studies showed that finite element model (FEM) combined to CAD/CAM improves the design of braces for the conservative treatment of adolescent idiopathic scoliosis (AIS), using 2D measurements from in-brace radiographs. We aim to assess the immediate effectiveness on curve correction in all three planes of braces designed using CAD/CAM and numerical simulation compared to braces designed with CAD/CAM only.

**Methods:**

SRS standardized criteria for bracing were followed to recruit 48 AIS patients who were randomized into two groups. For both groups, 3D reconstructions of the spine and patient’s torso, respectively built from bi-planar radiographs and surface topography, were obtained and braces were designed using the CAD/CAM approach. For the test group, 3D reconstructions of the spine and patient’s torso were additionally used to generate a personalized FEM to simulate and iteratively improve the brace design with the objective of curve correction maximization in three planes and brace material minimization.

**Results:**

For the control group (CtrlBraces), average Cobb angle prior to bracing was 29° (thoracic, T) and 25° (lumbar, L) with the planes of maximal curvature (PMC) respectively oriented at 63° and 57° on average with respect to the sagittal plane. Average apical axial rotation prior to bracing was 7° (T) and 9° (L). For the test group (FEMBraces), initial Cobb angles were 33° (T) and 28° (L) with the PMC at 68° (T) and 56° (L) and average apical axial rotation prior to bracing at 9° (T and L). On average, FEMBraces were 50% thinner and had 20% less covering surface than CtrlBraces while reducing T and L curves by 47 and 48%, respectively, compared to 25 and 26% for CtrlBraces. FEMBraces corrected apical axial rotation by 46% compared to 30% for CtrlBraces.

**Conclusion:**

The combination of numerical simulation and CAD/CAM approach allowed designing more efficient braces in all three planes, with the advantages of being lighter than standard CAD/CAM braces. Bracing in AIS may be improved in 3D by the use of this simulation platform. This study is ongoing to recruit more cases and to analyze the long-term effect of bracing.

**Trial registration:**

ClinicalTrials.gov, NCT02285621

## Background

Orthopedic bracing is the conservative treatment generally prescribed to control curve progression in adolescent idiopathic scoliosis (AIS) showing curves between 20° and 40° of Cobb angle [1]. AIS is a three-dimensional (3D) deformity of the spine which includes a deviation in the coronal plane, changes in the sagittal curves, and an axial rotation of the vertebrae [2, 3]. Bracing was demonstrated as an effective treatment to prevent curve progression, as assessed using 2D coronal X-ray measurements, and immediate in-brace correction was found to be correlated to long-term effectiveness [4–7]. The treatment outcomes rely on multiple factors such as timing with adolescent growth spurt, spine flexibility, and patient compliance to treatment [8–11].

However, bracing is not always successful and there is a lack of knowledge regarding the correction in the sagittal and transverse planes [12]. Studies reported that brace wear tends to create a hypokyphotic effect and provide a non-significant correction of vertebral axial rotation [13, 14], as well as having no effect on the orientation of the planes of maximum curvature (PMC), which are defined by the planes passing through the apex and the end vertebrae of a given curve [2, 13, 15].

### Traditional vs. CAD/CAM brace fabrication

Computer-aided design/computer-aided manufacturing (CAD/CAM) systems are now frequently used for brace design and have proven to be as effective compared to the traditional plaster-cast methods, using 2D metrics [16]. Traditional brace fabrication of rigid thoraco-lumbo-sacral orthosis (TLSO) is based on craftsmanship and involves plaster molding, which requires time and material consumption and presents a low accuracy [17]. However, the CAD/CAM design technique does not have an impact on brace effectiveness’ improvement. For this purpose, finite element models (FEM) have been developed to analyze brace biomechanics [18–23]. More recently, a simulation platform was created by combining CAD/CAM system and FEM [24, 25]. It allows the simulation of brace installation on the patient before its fabrication and the iterative improvement of its design and biomechanical efficiency [24, 25]. A randomized controlled trial (RCT) using this simulation platform was previously realized to evaluate the effectiveness of braces designed using this platform compared to standard braces designed with the plaster-cast technique and the CAD/CAM technology only [26]. Braces designed with the simulation platform were found to be more effective than the standard braces for correcting the major thoracic curve. Yet, measurements of brace effectiveness were done in 2D using the postero-anterior and lateral radiographs, and it remained to be demonstrated that the 3D correction of the curve could also be improved by the use of this simulation platform.

The objective of this study was to revisit the data from the previous RCT study to assess the 3D immediate effectiveness of braces designed using CAD/CAM and FEM compared to CAD/CAM only.

## Methods

### Study design

Inclusion criteria for this study were based on the SRS standardized criteria for bracing, and patients were consecutively recruited at our scoliosis clinic [27]. Inclusion criteria were AIS diagnosis (Cobb angle between 20° and 40°), a Risser sign of 0–2, and a full-time TLSO prescription. The study was approved by our institutional ethical committee, and each participant and their parents gave a written consent.

A simple randomization sequence was prepared by a biostatistician not involved in the recruitment and follow-up of the patients and was generated by a randomization table (simple block randomization list with a block size of 4). Patients were assigned to their group using the randomization sequence. The caregivers were blinded but not the orthotist. All patients had their brace designed by one of the two participating orthotists having more than 10 years of experience with TLSO and 2 years of experience with CAD/CAM technology. The patients from the control group received a TLSO designed and fabricated using the CAD/CAM approach only (CtrlBrace) while the patients from the test group received a TLSO designed and fabricated using the CAD/CAM approach but additionally simulated using a patient-specific FEM (FEMBrace).

### Brace design and fabrication

For all patients, simultaneous calibrated bi-planar postero-anterior (PA) and lateral (LAT) radiographs were taken during the patient’s first visit and after the brace installation using a low-dose digital radiography system (EOS™, EOS imaging, Paris, France). The 3D reconstruction was done with a custom-developed software using a semi-automated method based on 19 anatomical control points on the vertebral bodies, pedicles, and posterior arches [28]. The following indices were computed using the initial and in-brace 3D reconstructions using a custom measurement software: main thoracic (T) and lumbar (L) Cobb angles, kyphosis (T4–T12) and lordosis (L1–S1) angles, apical rotation at the apex of both T and L curves, and orientation of the PMC for T and L curves [29]. The precision using this software has been shown to be less than 1.5 mm for mean point-to-surface error and inferior to 5° for angular measurements compared to computed tomographic scan reconstructions [30].

For all patients, the external torso geometry was acquired using a surface topography system (3-dimensional Capturor, Creaform Inc., Levis, Canada) [31, 32]. Modification of the external torso geometry was done by the orthotists using a CAD/CAM software (Rodin4D, Bordeaux, France) to design the shape of the brace by virtually adding or removing material to introduce pressure and relief areas and corrective translations. Braces were then fabricated using a numerically controlled carver (Model C, Rodin 4D, Bordeaux, France) linked to the CAD/CAM software. A polyurethane foam bloc was carved according to the CAD model, and brace shell thermoforming was done using a heated copolymer sheet. The fabricated brace was trimmed and adjusted by the orthotist, and brace effectiveness was assessed using the 3D reconstruction of the spine obtained from calibrated PA and LAT in-brace radiographs [28]. The brace covering surface area was computed by importing the STL file of the brace design in a CAD/CAM software (CATIA V5R21, Dassault Sytemes, Vélizy-Villacoublay, France). Brace shell thickness and corrective pad thickness were measured by the orthotist following brace adjustment using a caliper tool [26].

### Additional steps for the test group (FEMBrace)

**Fig. 1:**
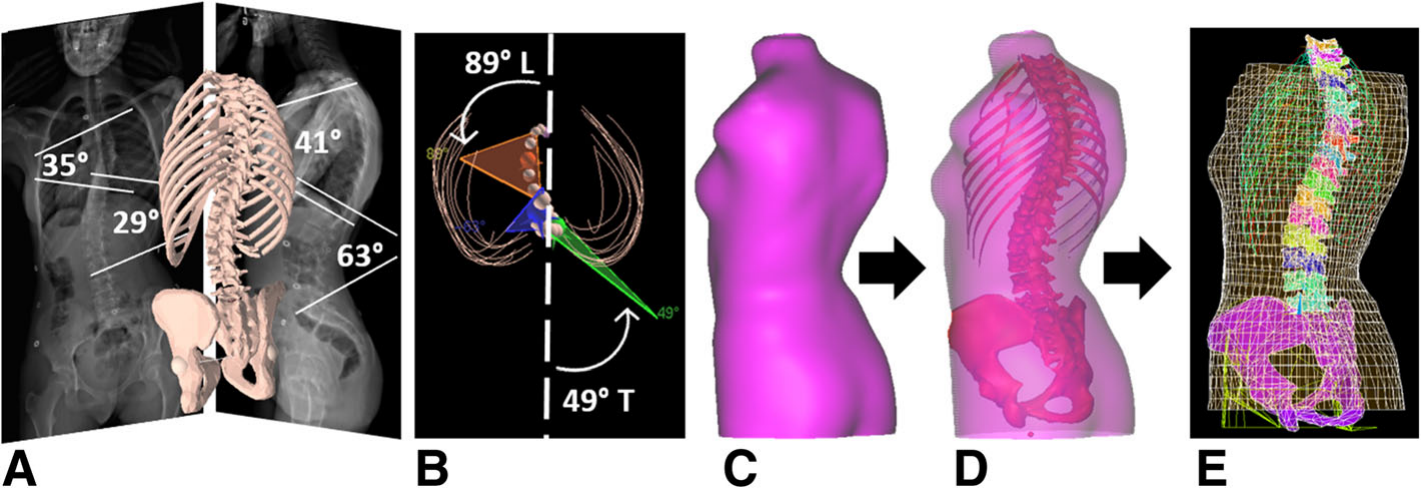
**a** Acquisition of the calibrated bi-planar radiographs and view of the corresponding 3D reconstruction of the spine, rib cage, and pelvis. **b** Top view of the planes of maximal curvature. **c** Torso 3D geometry following surface topography acquisition. **d** 3D geometric registration of the spine and torso geometry. **e** Finite element model of the trunk: vertebrae, intervertebral discs, ribs, sternum, costal cartilages, ligaments, and soft external tissues

Radiopaque markers visible on X-rays and trunk surface were a priori positioned on anatomical points of the patient’s torso (vertebrae T1 and L5, sternum jugular notch and xiphoid process, right and left anterior iliac spines) and were used to register the 3D reconstruction and the external torso geometry using a point-to-point least square algorithm. Using a previously validated method, the registered geometry was used to create a personalized FEM using Ansys 14.5 software package (Ansys Inc., Canonsburg, PA, USA) [7, 33]. The FEM includes the vertebrae T1 to L5, intervertebral discs, ribs, sternum, costal cartilages, ligaments, abdominal cavity, and soft external tissues (Fig. 1). Mechanical properties of the anatomical structures were taken from published data obtained on typical human cadaveric spine segments [20, 21, 33–37].

**Fig. 2:**
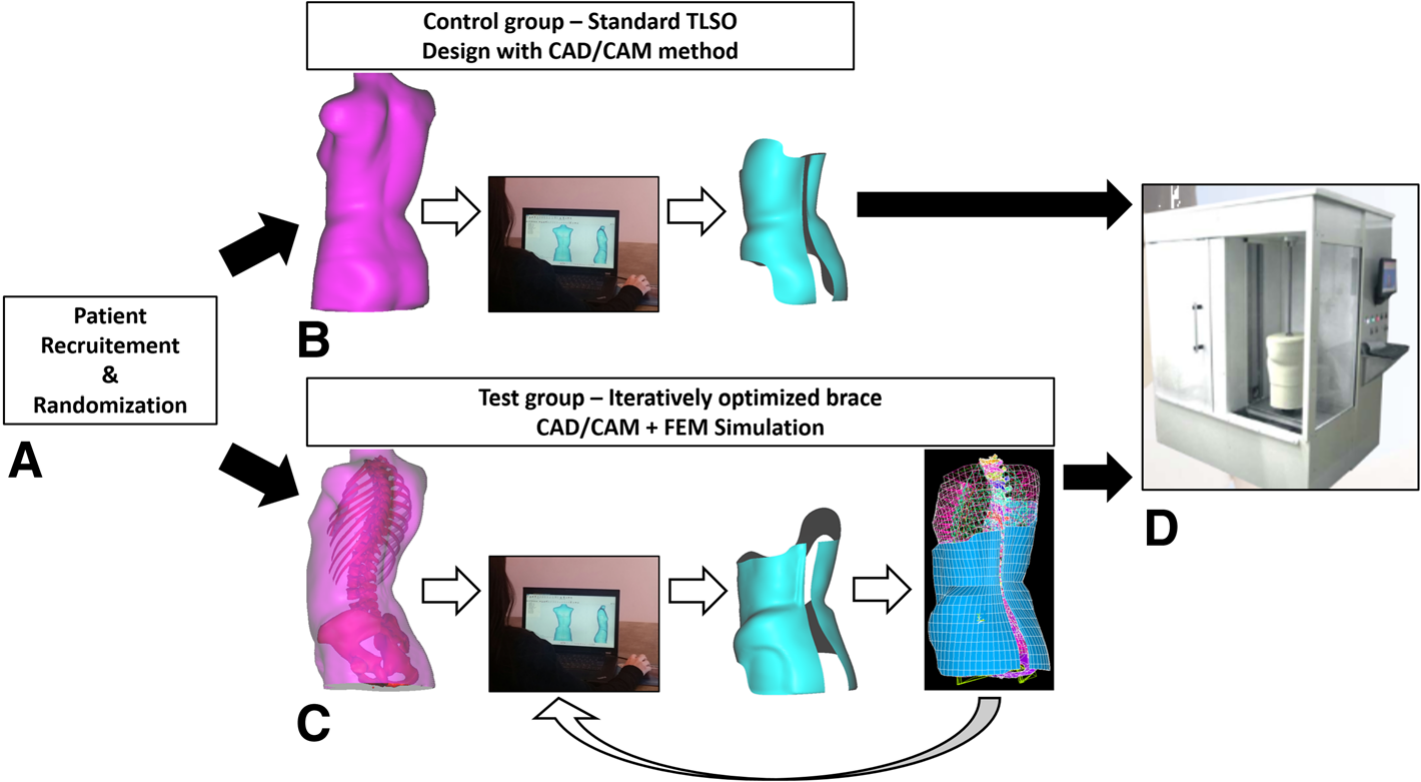
**a** Patient’s recruitment and randomization. **b** CtrlBrace design using the CAD software. **c** Iterative FEMBrace design using the CAD software and simulation of the FEMBrace installation. **d** Brace fabrication using a numerically controlled carver

For FEMBraces, during the brace design process, the 3D reconstruction of the initial patient’s spinal geometry (previously obtained for the FEM generation) was available and imported as an STL file in the CAD/CAM software to help position the corrective translation and pressure areas on the torso geometry. A brace FEM using polyethylene mechanical properties was then created. The orthotist selected nodes on the brace FEM to define the strap localization and virtually positioned the brace on the patient’s FEM. The brace installation was then simulated using a point-to-surface contact interface between the brace and the trunk models to represent friction and force transfer from the brace shell to the patient’s trunk surface [33, 38]. During the brace installation simulation, the brace was opened by applying displacement on nodes of the brace posterior opening. The brace was then placed on the trunk model, and sets of co-linear forces were applied at the strap fixation sites as previously determined by the orthotist [33]. During all simulation steps, the pelvis was fixed in space and the first thoracic vertebra (T1) was allowed to rotate and translate longitudinally. For a given simulation, the correction was assessed using post-processed Cobb angles, lordosis and kyphosis, vertebral axial rotation, and the orientation of the PMC, as well as the distance between patient’s skin and brace shell (brace fitting) (Fig. 2). Following the brace simulation, it was possible to modify the brace design to improve brace correction. To improve 3D correction, the brace design was iteratively modified by the orthotist in the CAD/CAM software by varying mainly the corrective pressure area localization and depth, as well as the trim lines, relief zones, side of trochanteric pressure area, and openings on the brace, and was simulated. The brace effectiveness was computationally assessed by the orthotist to maximize the correction using post-processed 3D indices. The strategy for correction maximization was to incrementally accentuate pad depth by 5 mm until simulated spinal correction remained stable even with the corrective area depth increasing (2° Cobb angle) [25]. The numerical process required an average of 3 iterations per patient (minimum 2, maximum 6). The strategy for minimizing the brace surface contact was to create openings in the brace shell at locations where the simulated distance between brace material and patient’s skin was more than 6 mm.

The optimal FEMBrace was then fabricated using the same numerical controlled carver and thermoforming process as for the control group (CtrlBrace). The FEMBrace was trimmed by the orthotist, and brace effectiveness was assessed using the 3D reconstruction of the spine computed from simultaneous PA and LAT radiographs. Brace covering surface and brace thickness were measured using the same methods as for CtrlBrace.

### Statistical analysis

Statistical analysis was performed using STATISTICA 10.0 software package (Statistica, StatSoft Inc., Tulsa, Oklahoma, USA). To verify that both groups were statistically comparable, a paired Student *t* test (95% significance level) was applied to compare the curve severity, T4–T12 kyphosis, L1–S1 lordosis, apical axial rotation, and the orientation of the PMC between both groups. A statistical analysis was also realized using a paired Student *t* test (95% significance level) to analyze if there was a significant difference between both groups for in-brace indices.

## Results

Twenty-five patients and 23 patients were respectively recruited in the control group and the test group. Following statistical analysis, both groups were found comparable and had non-statistically different age, sex, weight, height, skeletal maturity, curve type, curve severity, and initial 3D parameters (Table 1). For the control group, average Cobb angle prior to bracing was 29° (T) and 25° (L) and the apical axial rotation was of 7° for the T curve and 9° for the L curve with respective PMC oriented at 63° and 57° with respect to the sagittal plane. For the test group, average Cobb angle prior to bracing was 33° (T) and 28° (L) and the average apical axial rotation was of 9° for both T and L curves, with respective PMC of 68° and 56°. For both groups, average initial T4–T12 kyphosis was 25° and average L1–S1 lordosis was 66°.

**Table 1:** Patient data at initial visit (measurements in the three planes computed using the 3D reconstruction of the spine)

		Test group *N* = 25			Difference between groups	CtrlBrace *N* = 23		
		Mean	SD	*N*	Paired *t* test^a^	Mean	SD	*N*
Coronal plane	T Cobb angle	33°	8°	23	*p* = 0.06	29°	8°	20
	L Cobb angle	28°	9°	21	*p* = 0.14	25°	10°	17
Transverse plane	T apical axial rotation	9°	3°	23	*p* = 0.41	7°	6°	20
	L apical axial rotation	9°	5°	21	*p* = 0.60	9°	6°	17
	T plane of maximum curvature (angle with respect to the sagittal plane)	68°	17°	23	*p* = 0.28	63°	23°	20
	L plane of maximum curvature (angle with respect to the sagittal plane)	56°	19°	21	*p* = 0.66	57°	26°	17
Sagittal plane	T4–T12 kyphosis	25°	15°	25	*p* = 0.91	25°	12°	23
	L1–S1 lordosis	66°	9°	25	*p* = 0.91	66°	11°	23

^a^Statistically significantly different for *p* < 0.05

The coronal plane correction was statistically significantly greater in the test group vs. the control group (*p* < 0.05): FEMBraces reduced T Cobb angle by 47% while it reduced the L Cobb angle by 48% vs. 25 and 26% respectively for the CtrlBraces (Table 2). The actual FEMBrace Cobb angle correction was predicted with an average difference inferior to 5° by the simulation.

**Table 2:** In-brace results for T and L Cobb angles, apical axial rotation for T and L apex, and orientation of the planes of maximal curvature and kyphosis and lordosis angles

		FEMBrace (test group)		Difference between groups	CtrlBrace (control group)	
		Mean	SD	Student’s *t* test^a^	Mean	SD
Coronal plane	T Cobb angle reduction (%)	47	20	*p* = 0.01	25	18
	L Cobb angle reduction (%)	48	24	*p* = 0.04	26	27
Transverse plane	T curve apical axial rotation correction (%)	46	24	*p* = 0.004	30	17
	L curve apical axial rotation correction (%)	46	22	*p* = 0.003	30	23
	T plane of maximum curvature reduction (degrees)	0	18	*p* = 0.28	3	30
	L plane of maximal curvature reduction (degrees)	11	40	*p* = 0.66	11	46
Sagittal plane	T4–T12 kyphosis reduction (degrees)	−2	6	*p* = 0.02	−16	28
	L1–S1 lordosis reduction (degrees)	12	25	*p* = 0.44	11	24

^a^Significant difference between both groups for *p* < 0.05

In the transverse plane, the correction also was statistically significantly greater in the test group vs. the control group (*p* < 0.05): apical axial rotation was corrected by 46% for FEMBraces vs. 30% for CtrlBraces for both T and L curves (Table 2). A statistically significant corrective effect was found between the in-brace-corrected PMC and the out of brace initial PMC for the L curve (*p* value = 0.01) for both groups. However, the orientation of the PMC of the T curve was not really modified in both groups, but with larger variability in the control group.

In the sagittal plane, the kyphosis was significantly less reduced in the test vs. control group (*p* < 0.05) (2 vs. 16%), but there was no significant difference for the change of lordosis (21 vs. 16%) (Table 2). Detailed statistical results including the *p* values are described in Table 2.

**Fig. 3:**
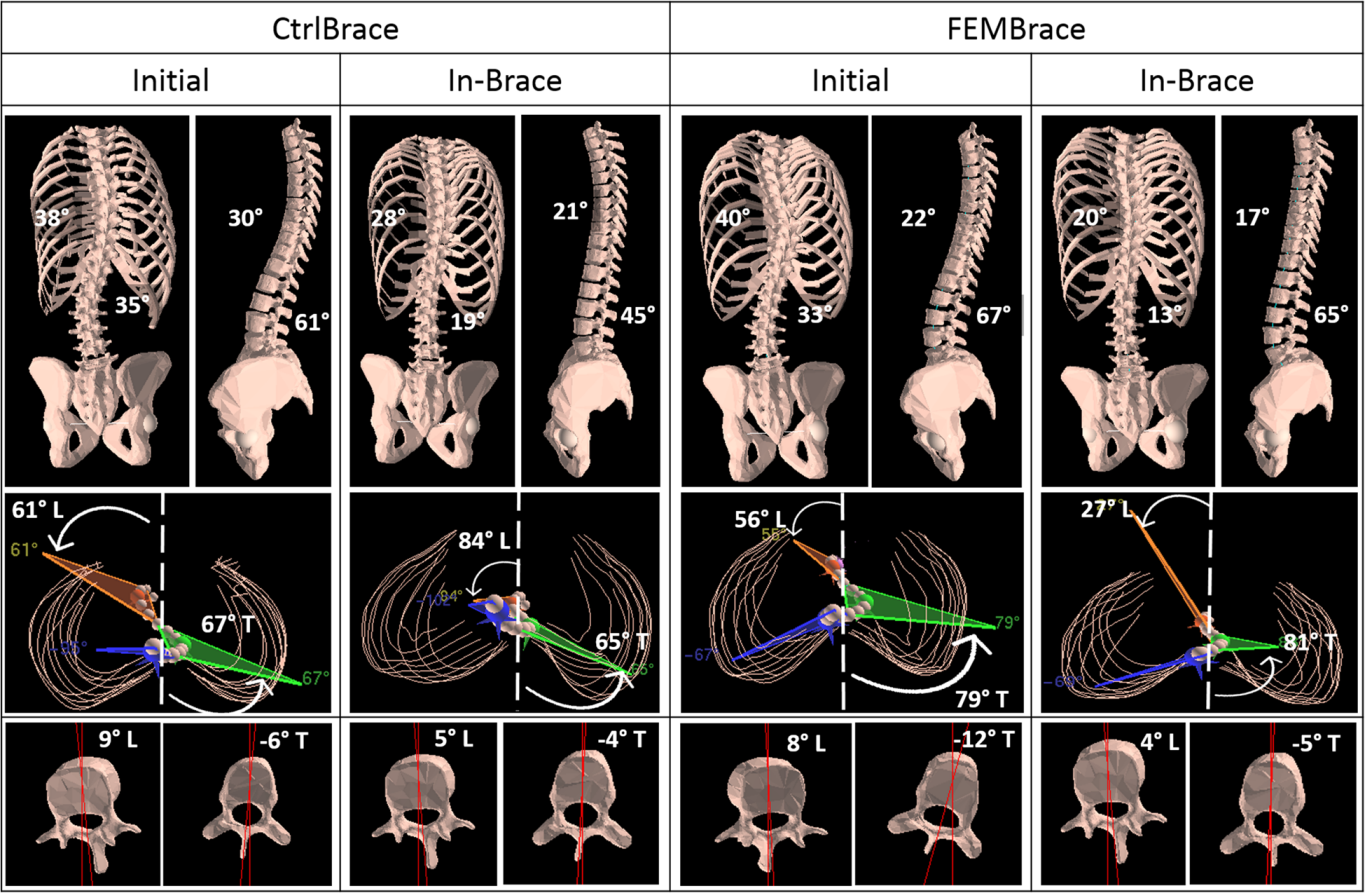
Results in the coronal (T and L Cobb angles), sagittal (kyphosis and lordosis), and transverse planes (T and L PMC as well as T and L apical axial rotation) for two typical patients: out of brace initial curve, with the CtrlBrace or with the FEMBrace

FEMBraces had an average of 20% less covering surface than the CtrlBraces. The brace shell thickness was the same for both groups (4 mm), but 13-mm-thick pads were added to the CtrlBraces (no foam pad (liner) was necessary for the FEMBraces). As CtrlBrace foam pads were covering on average 34% of the brace area, we estimated globally FEMBraces to be 50% thinner than CrtlBraces (Fig. 3).

The time needed for the orthotists to complete the iterative brace design process was of 5 min to start the simulation (manipulations to prepare and position the brace model on the trunk model, selection of the strap fixation sites) and 10–15 min per iteration to perform the modifications on the brace design in the CAD/CAM software for the next simulation. However, the time needed for the brace fitting was reduced by approximately 30 min as compared to the CtrlBraces.

## Discussion

This study demonstrated a clinically and statistically significant greater 3D immediate in-brace effectiveness for braces designed using a new design platform combining CAD/CAM and FEM compared to CAD/CAM only. The main strength of this study is the 3D analysis and the RCT design, which confirms and supports previous feasibility studies with CAD/CAM and FEM simulations for brace design in AIS. It distinguishes from previous studies for which brace effectiveness was only evaluated in 2D using only the PA and LAT radiographs.

Using this simulation platform, it is possible to simulate/test different brace designs and better define the treatment plan to include the sagittal and transverse plane correction parameters. We believe that having access to spinal 3D reconstructions and FEM combined with CAD/CAM techniques allows orthotists to better visualize and address the sagittal, transverse, and coronal profiles of the spine. This could improve brace design with better 3D fitting to correct efficiently the spinal deformity in the frontal plane, as well as in the transverse plane, while preserving the kyphosis and lordosis curvatures in the sagittal plane.

Furthermore, by performing the measurements using the 3D reconstruction instead of radiographs, it was possible to assess additional 3D parameters, which were not evaluated in previous studies [24–26] such as the axial rotation at the apex of the curves and the orientation of the PMC for both T and L curves. Vertebral axial rotation is possibly associated with curve progression [39], and correcting or controlling this parameter could improve brace effectiveness for the long-term results.

The limited action of the CtrlBraces on the orientation of the PMC was also reported in previous studies [13, 37]. The PMC combines the regional description of the spine curvature in both the sagittal and the coronal planes; therefore, it is not an independent index as compared to the vertebral axial rotation, which is a measurement of the mechanical torsion in the spine. The components of brace design that could address the residual regional deformity of the PMC remain to be dealt with. The use of the patient-specific FEM could be useful to further improve the 3D effectiveness of braces.

However, there are limitations to this trial. The detailed muscles and muscular activation were not modeled in the FEM but were indirectly represented through a global evaluation of forces required to maintain the balance at T1. In this study, we only addressed the immediate effect of wearing a brace. Since a correlation has been reported between immediate in-brace correction and brace treatment long-term effectiveness [5] and that 3D parameters related to curve progression seem to be better controlled, it suggests that FEMBraces may also improve the long-term treatment efficacy in all three planes. A study of long-term effects in a larger cohort appears warranted to evaluate if the 3D correction of the deformity influence the treatment’s outcomes.

The use of the simulation platform allowed orthotists to analyze the contact surface between brace and patient’s skin, in order to adjust the openings and relief zones on the brace to obtain less covering surface and thinner braces. The addition of FEM to CAD/CAM techniques was not more time-consuming and did not add complexity to brace fabrication as the brace was optimized.

This simulation platform allowed to test any rigid brace design; therefore, it could also be used to study or improve any other braces like the ones with an anterior opening, orthoses used to treat scoliotic thoraco-lumbar curves or orthoses presenting possible 3D spinal correction in the sagittal and the transverse planes [40].

## Conclusions

Combining the CAD/CAM approach with FEM simulation allowed the design of more efficient braces to correct the scoliotic spinal deformities in all three planes at the first immediate in-brace evaluation, with lighter design than standard CAD/CAM braces. These results suggest that long-term 3D effect of bracing in AIS may be improved by the use of this new platform, but this should be further tested as part of an ongoing RCT study. We feel that the ability to assess the biomechanical effects of bracing in 3D is becoming important.

## Abbreviations

3D: Three-dimensional
AIS: Adolescent idiopathic scoliosis
CAD/CAM: Computer-aided design/computer-aided manufacturing
FEM: Finite element model
L: Lumbar
LAT: Lateral
PA: Postero-anterior
PMC: Plane of maximum curvature
RCT: Randomized controlled trial
T: Thoracic
TLSO: Thoraco-lumbo-sacral orthosis
